# A Fuzzy Logic Model for the Analysis of Ultrasonic Vibration Assisted Turning and Conventional Turning of Ti-Based Alloy

**DOI:** 10.3390/ma14216572

**Published:** 2021-11-01

**Authors:** Riaz Muhammad

**Affiliations:** Mechanical Engineering Department, College of Engineering, University of Bahrain, Isa Town P.O. Box 32038, Bahrain; rmuhammad@uob.edu.bh

**Keywords:** fuzzy logic, turning, Ti-alloys, chip formation

## Abstract

Titanium and its alloys are largely used in various applications due its prominent mechanical properties. However, the machining of titanium alloys is associated with assured challenges, including high-strength, low thermal conductivity, and long chips produced in conventional machining processes, which result in its poor machinability. Advanced and new machining techniques have been used to improve the machinability of these alloys. Ultrasonic vibration assisted turning (UVAT) is one of these progressive machining techniques, where vibrations are imposed on the cutting insert, and this process has shown considerable improvement in terms of the machinability of hard-to-cut alloys. Therefore, selecting the right cutting parameters for conventional and assisted machining processes is critical for obtaining the anticipated dimensional accuracy and improved surface roughness of Ti-alloys. Hence, fuzzy-based algorithms were developed for the ultrasonic vibration assisted turning (UVAT) and conventional turning (CT) of the Ti-6Al7Zr3Nb4Mo0.9Nd alloy to predict the maximum process zone temperature, cutting forces, surface roughness, shear angle, and chip compression ratio for the selected range of input parameters (speed and depth-of-cut). The fuzzy-measured values were found to be in good agreement with the experimental values, indicating that the created models can be utilized to accurately predict the studied machining output parameters in CT and UVAT processes. The studied alloy resulted in discontinued chips in both the CT and UVAT processes. The achieved results also demonstrated a significant decline in the cutting forces and improvements in the surface quality in the UVAT process. Furthermore, the chip discontinuity is enhanced by the UVAT process due to the higher process zone temperature and the micro-impact imposed by the cutting tool on the workpiece.

## 1. Introduction

The balanced set of mechanical properties, lightweight, and corrosion to resistivity of titanium alloys make it an excellent choice for bellicose environment applications [1]. However, the high strength and low thermal conductivity causes severe challenges for mechanists in industries, resulting in poor surface quality, a high rejection rate, and tool wear [2]. Additionally, the spring back effect and continuous chip production of titanium alloys results time loss in production systems.

In the past, experiments and simulations have been used to study and improve the machinability of hard-to-cut alloys. These improvements were achieved by enhancing the machining capabilities [3,4,5,6], hybrid machining techniques [7], cryogenic coolant application [8], minimum lubricant quantity [9], and alloy modification without compromising the mechanical properties of the alloy [10]. The theory of ultrasonic machining has been successfully applied to face milling and drilling operations, and significant improvements in terms of the surface quality of the finished product has been achieved [11,12,13].

With these advancements in simulation tools, various studies have been conducted on the machining of titanium alloys [14]. Simulation tools allow readers to obtain the required results without spending time on costly and time-consuming experimental techniques. Many two- and three-dimensional finite element (FE) models have been developed for the orthogonal turning of Ti alloys to examine the outcomes of materials at several machining input parameters [15,16,17,18]. Similarly, with advances in computational facilities and software, three-dimensional FE simulation models have been established for conventional and assisted oblique turning processes [7,19].

With Industrial Revolution 4.0 and the advancement in CNC machines, most industries are moving toward the uninterrupted automatic machining of high-quality products. The long continuous chips produced in Ti alloys interrupt the machining time due to their entanglement with either the workpiece or cutting tool, affecting the surface quality of the finished product and the sharpness of the cutting tool, respectively. Therefore, artificial intelligence (AI) methods are becoming more desirable for the modeling of machining processes. Recently, a fuzzy-logic technique based on the combination of multivalued logic and the theory of probability to model complex engineering problems is gaining popularity in the research community [20]. Fuzzy-logic offers additional good judgment and concrete means to overcome the problem of commanding reasoning abilities confined by rules [21]. This method has been used by several investigators for the prediction of machining results based on the various input machining parameters as listed in Table 1.

Ultrasonic vibration-assisted turning (UVAT) is a machining technique in which vibrations are imposed on the cutting insert, resulting in the intermittent cutting of materials [38]. UVAT has shown significant improvements in surface quality and a decline in the cutting forces in hard-to-cut materials [19,39,40]. In the current work, a fuzzy logic technique is adopted for the simulation of UVAT and the conventional turning (CT) processes of Ti-6Al7Zr3Nb4Mo0.9Nd to predict the cutting forces, maximum process zone temperature, chip compression ratio (CCR), surface roughness, and shear angle (SA). The fuzzy model was validated with experimental results and was used for the comparative analysis of UVAT and CT.

## 2. Proposed Fuzzy Logic Algorithm

### 2.1. Fuzzy-Based Algorithms for UVAT and CT

**Figure 1:**
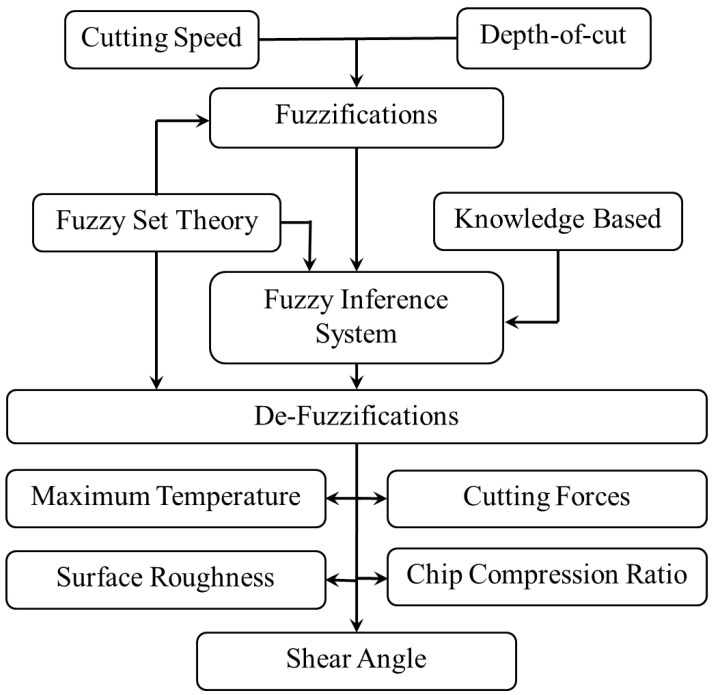
Schematic illustration of developed fuzzy inference systems for CT and UVAT.

Figure 1 presents the conceptual illustration of the developed fuzzy inference systems for the calculation of the maximum process zone temperature, cutting forces, surface roughness, CCR, and SA in CT and UVAT at the selected range of input parameters.

The developed fuzzy inference systems are designed for two input variables, speed (V) and depth-of-cut (DoC), for the prediction of output variables such as maximum temperature, cutting forces, surface roughness, CCR, and SA during the CT and UVAT processes. The input variables are fed to the fuzzy plane, and the selected output variables are determined based on the defined fuzzy rules-based system. Moreover, in the developed algorithms, the output calculation of the scheme is established on the centroid-method, and de-fuzzification is accomplished using the Mamdani implication. The feed rate used in the current simulations were assumed to be constant at 0.1 mm/rev, whereas the axial force (*Fa*) component was also ignored due to its low level when compared to the radial force (*Fr*) and tangential force components (*Ft*). An amplitude of 10.0 µm and a frequency of 20.0 kHz were assumed in the developed UVAT process.

#### 2.1.1. Fuzzy Membership Functions

A triangular membership function was used for the input (V and DoC) and output (tangential/radial forces, maximum process zone temperature, surface roughness, CCR, and SA) variables in the developed models based on the recommendation of Pedrycz [41]. The triangular function is mathematically represented in Equation (1):

(1) Variables(u,m,v)={0  s≤us−um−s  u<s≤mv−sv−m  m<s≤v0  s≥v

where *u*, *m*, and *v* represent triangular membership function. Details can be found elsewhere [21].

#### 2.1.2. Fuzzy Sets Used in Simulation

**Figure 2:**
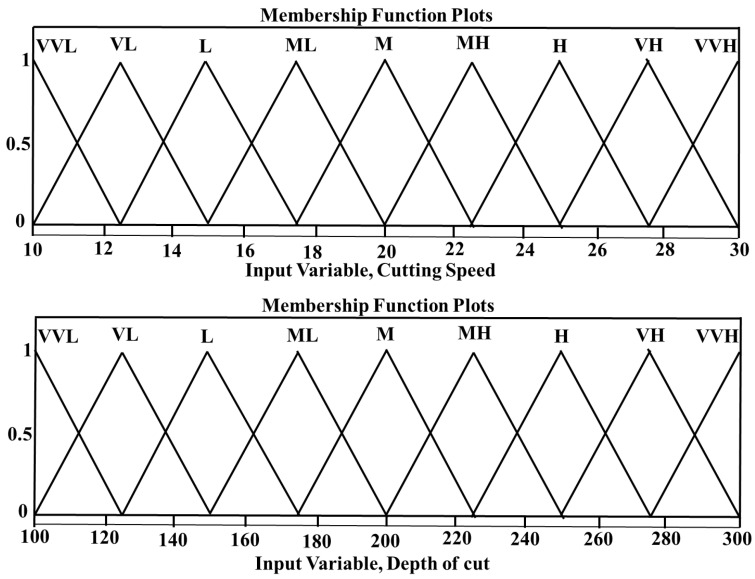
Input variables with associated membership functions used in the simulation of UVAT and CT.

**Figure 3:**
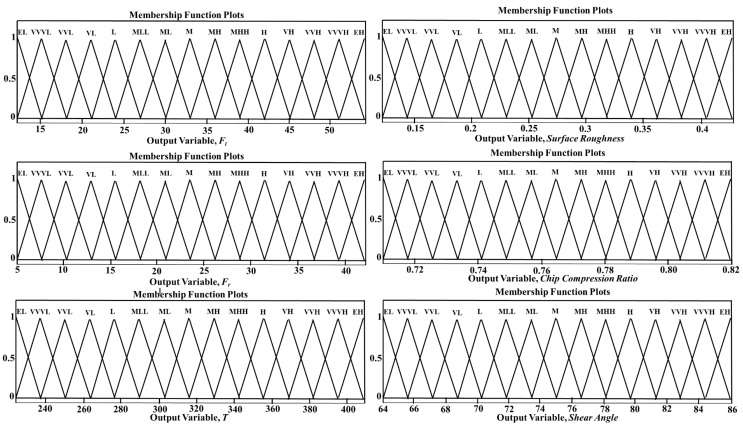
Output variables with associated membership functions used in the simulation of UVAT.

**Figure 4:**
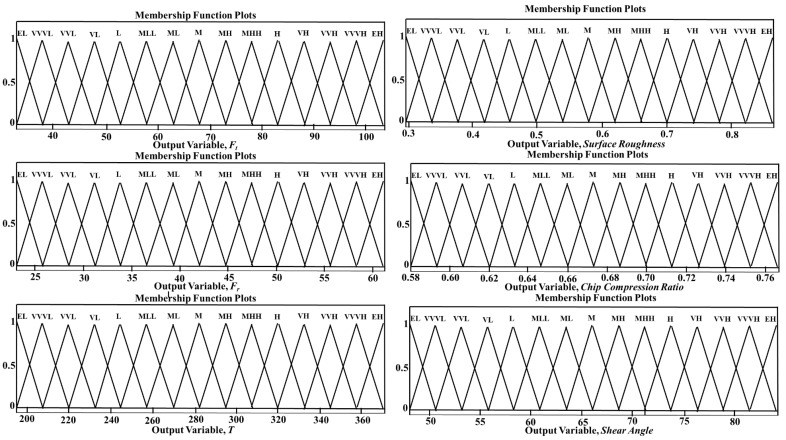
Output variables with associated membership functions used in the simulation of CT.

The two input variables V and DoC were divided into 9 fuzzy sets, which were represented as very-very-low (VVL), very-low (VL), low (L), medium-low (ML), medium (M), Medium-high (MH), high (H), very-high (VH), and very-very-high (VVH) in both the CT and UVAT in the developed fuzzy inference systems. The feed rate in the developed models was assumed to be constant at 0.1 mm/rev. Additionally, the output variables were distributed into 16 fuzzy-sets, i.e., extremely-low (EL), very-very-very-low (VVVL), very-very-low (VVL), very-low (VL), low (L), medium-low-low (MLL), medium-low (ML), medium (M), medium-high (MH), medium-high-high (MHH), high (H), very-high (VH), very-very-high (VVH), very-very-very-high (VVVH), and extremely-high (EH). The defined input variables with their related membership functions are given in Figure 2. The output variables for the UVAT and CT systems with their associated membership functions are presented in Figure 3 and Figure 4, respectively. Additionally, additional information and terminology considered for the defined input and output fuzzy sets used in the simulations of the CT and UVAT processes are listed in Table 2 and Table 3, respectively.

#### 2.1.3. Proposed Rules

The set of rules were defined for both the developed fuzzy inference systems to calculate the output variables against the defined combinations of the input variables within the defined range, as represented in Table 4 and Table 5 for UVAT and CT, respectively.

## 3. Experimental Work

A Ti-6Al-7Zr-3Nb-4Mo-0.9Nd designated as a T-6734-0.9Nd rod that had a length of 90 m and a diameter of 25 mm was received from the Technical University of Braunschweig, Germany. A thermosetting adhesive was used to join the received small piece of Ti-alloy to a mild steel specimen to be able to mount it in the chuck of a Lathe machine. DNMG 150608 MF1 CP500 inserts were used in the experiments, and details can be found elsewhere [3,7].

Three cutting speeds of 10, 20, and 30 m/min and three DoC levels (100, 200, and 300 µm) were selected for the experiments to validate the predicted simulation results. Each set of experiments was repeated three times, and no cutting fluid was used in the tests.

A modified universal Harrison M-300 Lathe machine was used to conduct the experiments on the as received alloys for the CT and UVAT process. The cutting forces were measured using a force sensor (KIAG-SWISS/Type9257A) made by Kistler. The axial force component was ignored during experimentation due to its low intensity compared to *Ft* and *Fr*. The frequency and amplitude were set to 20 kHz and 10 um, respectively, in the UVAT process. A calibrated FLIR (ThermaCAMTM SC-3000) thermal system was used for the measurement of the maximum process-zone temperature in CT and UVAT. The quick view specialized software was used for the analysis of the results.

The surface quality assessments of the machined samples were conducted on a Zygo^®^-newview-5000 interferometer. The *Ra* was assessed at the tested cutting conditions, and data were taken at various locations. The chips produced at various cutting conditions were collected and analyzed for CCR and SA in UVAT and CT. The chip collected at 100 µm and higher speeds were hard to mount in Bakelite resin and were excluded from experimentation. Additionally, the metallographic analysis of the alloy is not included in the current work.

## 4. Results and Discussion

### 4.1. Simulations Results

The commercially available simulation tool MATLAB 2014b was used for the developed algorithms based on the fuzzy logic inference system for UVAT and CT. The developed models were able to predict *Fr*, *Ft*, process zone temperature, surface roughness, CCR, and SA at various speed and depth-of-cut (DoCs) combinations as in input parameters.

**Figure 5:**
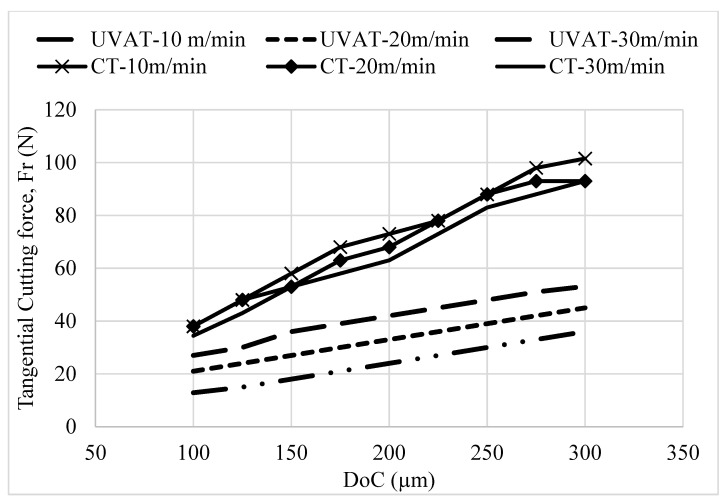
The predicted *Ft* in CT and UVAT.

**Figure 6:**
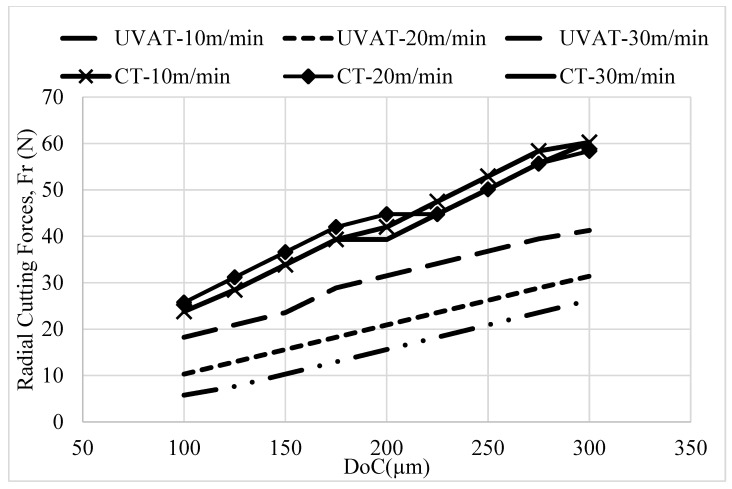
The predicted *Fr* in CT and UVAT.

The predicted *Ft* and *Fr* at selected cutting speeds and DoCs in CT and UVAT are presented in Figure 5 and Figure 6, respectively. The models predicted the cutting forces at various cutting parameters based on the rules defined and are consistent with the previously published work on UVAT and CT processes [3,10,38,42]. The model developed for UVAT process predicted a lower average value of *Ft* and *Fr* at various combinations of speed and DoCs when compared to the CT results.

A gradual increase in the predicted *Ft* and *Fr* was observed, with an increase in the speed and DoC during the UVAT process, as expected. Similarly, the model developed for CT foretold a gradual increase in the cutting forces, with a rise in the DoC. However, a minor variation in the *Ft* and *Fr* at selected cutting speeds was achieved in CT and was in good agreement with the findings of Muhammad et al. [3,7]. An average decline of approximately 60–70% in the cutting forces was described by the simulation of the UVAT process when compared to CT at lower tested cutting speeds.

**Figure 7:**
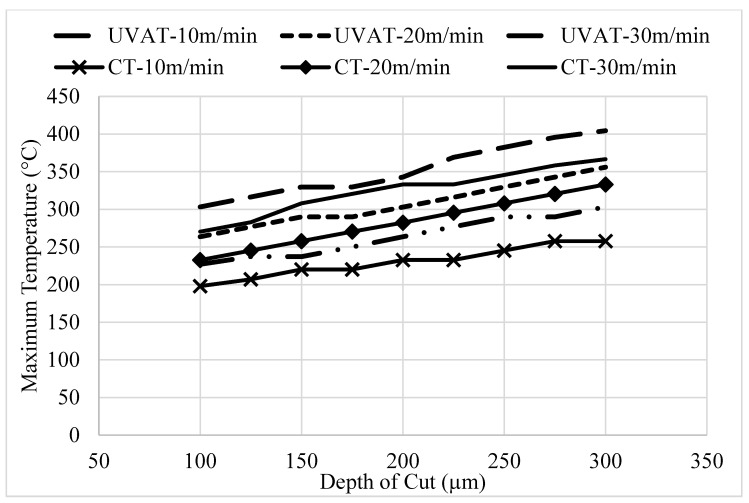
The predicted maximum temperature results in UVAT and CT.

The maximum temperature predicted in the UVAT and CT processes by the developed models is shown in Figure 7. A gradual increase in the maximum temperature with the cutting speed and DoC was measured in during the UVAT and CT processes [10,42]. However, the temperature level predicted in UVAT was slightly higher when compared to the temperature level predicted for CT due to the additional increase in the relative velocity due to vibration coupling on the cutting insert, which is in good agreement with Naseer et al. [43].

**Figure 8:**
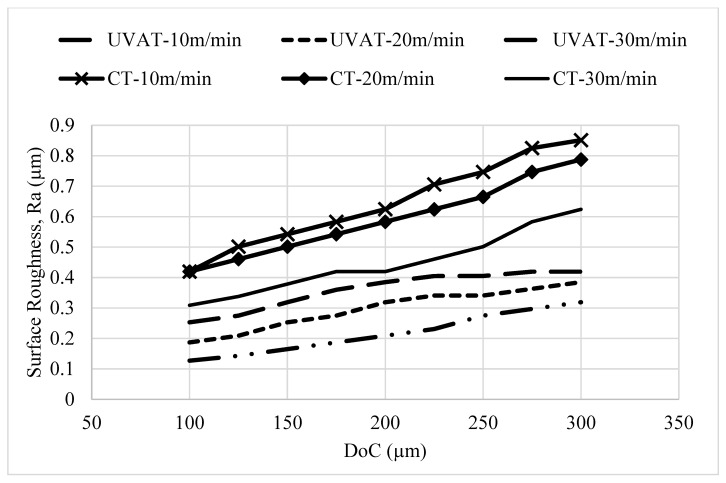
The predicted *Ra* in UVAT and CT.

The developed simulation models were utilized to predict the surface roughness parameter (*Ra*) for the tested combinations of speed and DoC, as demonstrated in Figure 8. The model predicted a significant improvement in *Ra* due to the ironing effect of the cutting insert on the machine surface. Similarly, the models developed for the UVAT and CT processes predicted an improvement in the surface quality with a growth in cutting speed, which is in good agreement with work of Silberschmidt et al. [44].

**Figure 9:**
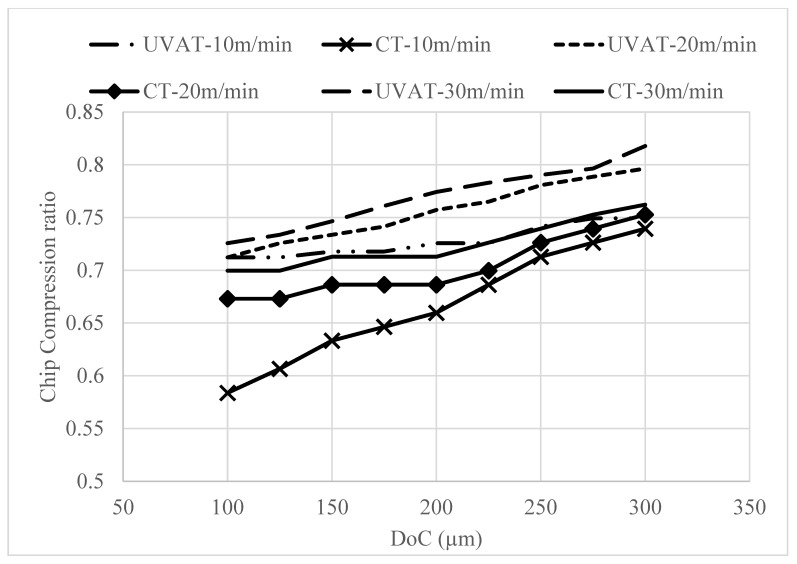
Predicted level of CCR in UVAT and CT.

**Figure 10:**
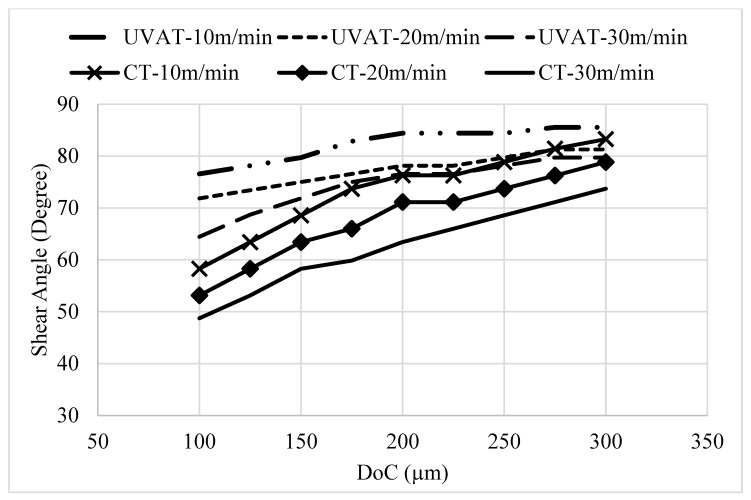
The simulated SA variation with speed and DoC in UVAT and CT.

The developed model was also used to predict the CCR and SA of the formed chips at various speed and DoC combinations. The predicted levels of CRR and SA are presented in Figure 9 and Figure 10, respectively. The models predicted a gradual increase in the CCR, showing growth in the speed and DoC in the studied processes [45]. However, the CCR in UVAT is significantly higher when matched to the CCR in CT.

A gradual rise in the SA was predicted in the CT and UVAT processes with DoC. However, with a growth in speed, a slight drop in the shear angle was noticed, which is in good agreement with [45].

### 4.2. Experimental Results

**Figure 11:**
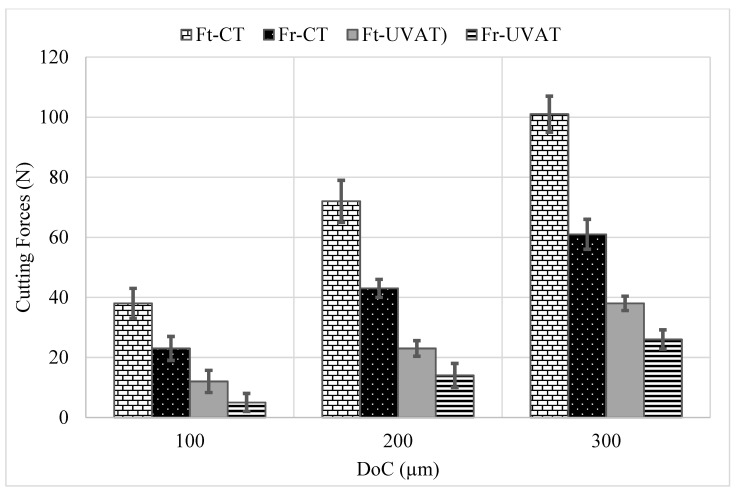
Cutting forces at various DoCs and constant speed of 10 m/min.

The experimental results demonstrate a significant decline of approximately 60–70% in the cutting forces in UVAT, as shown in Figure 11. At lower cutting speed of 10 m/min, the levels of *Ft* observed in CT at 100, 200, and 300 µm DoCs were 38, 72, and 101 N, respectively. Similarly, 23, 43, and 61 N forces in the radial direction were recorded for the same cutting conditions. On the other hand, a significantly lower level of *Ft* was measured in the UVAT process. The effect of vibrations on cutting forces is substantial at lower cutting speeds due to longer separation between the insert edge and chip [17].

**Figure 12:**
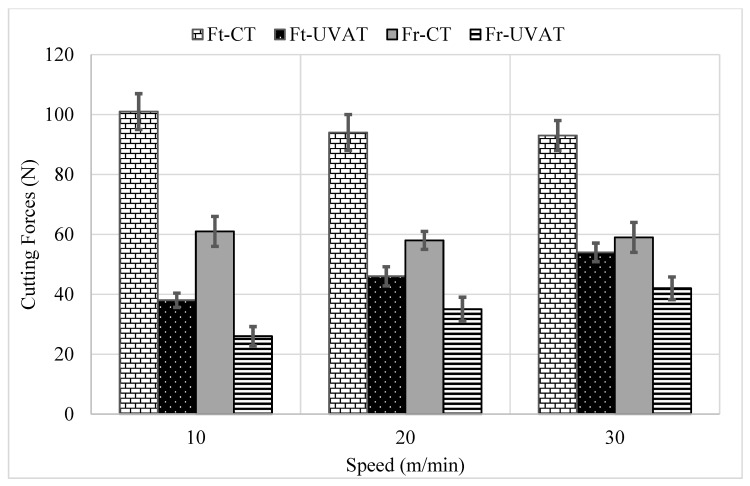
Experimentally measured cutting forces at various speeds and DoC = 300 µm.

The cutting forces were measured at various cutting speeds in CT, and it was found that speed has no significant effect on it (see Figure 12). However, in the UVAT process, a gradual increase in *Ft* and *Fr* was recorded with an increase in cutting speed. The *Ft* merely increased from 38 to 54 N when the speed was increased from 10 to 30 m/min and DoC = 300 µm. The same trend was also seen at lower DoCs.

**Figure 13:**
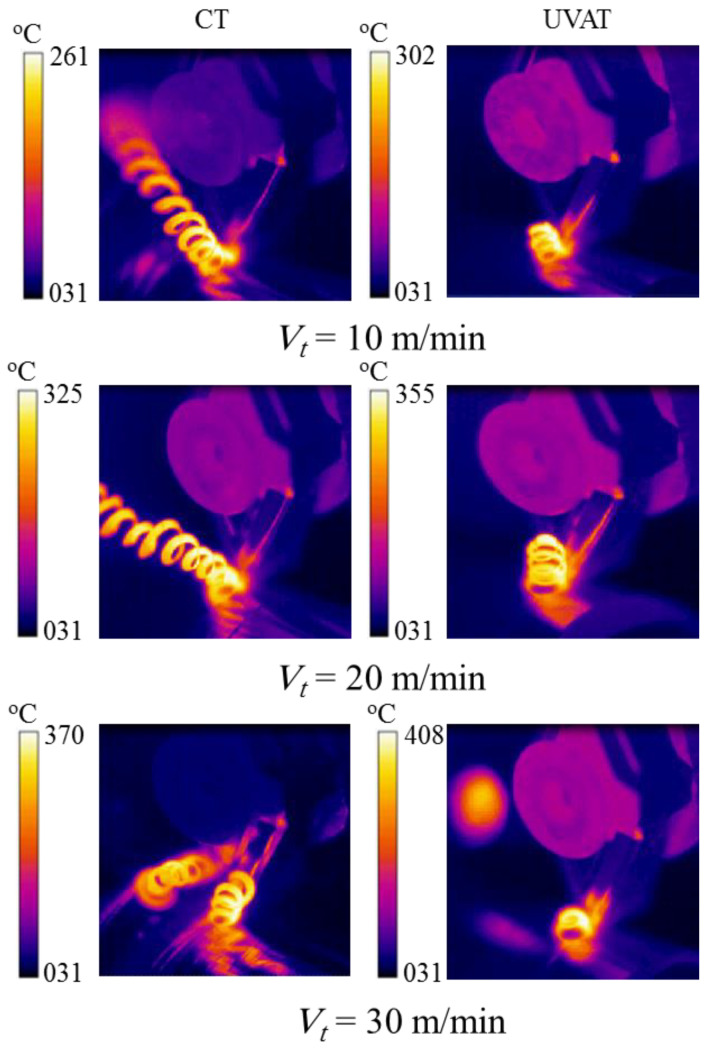
Measured temperature in CT and UVAT.

The maximum temperatures recorded during the CT and UVAT processes are shown in Figure 13. A slightly higher temperature level was recorded in UVAT (408 °C) when matched to CT (327 °C) at the 30 m/min cutting speed and the 300 µm DoC. The same trend was observed in all of the tested cutting conditions. The increase in temperature can be linked with escalation in the relative velocity of the tool in one complete vibrational cycle and with the additional power provided to the cutting edge in the form of vibration [7,42,43]. The same trend was observed when the DoC was increased as well; however, the impact of speed is foremost, as expected [39,40,46].

**Figure 14:**
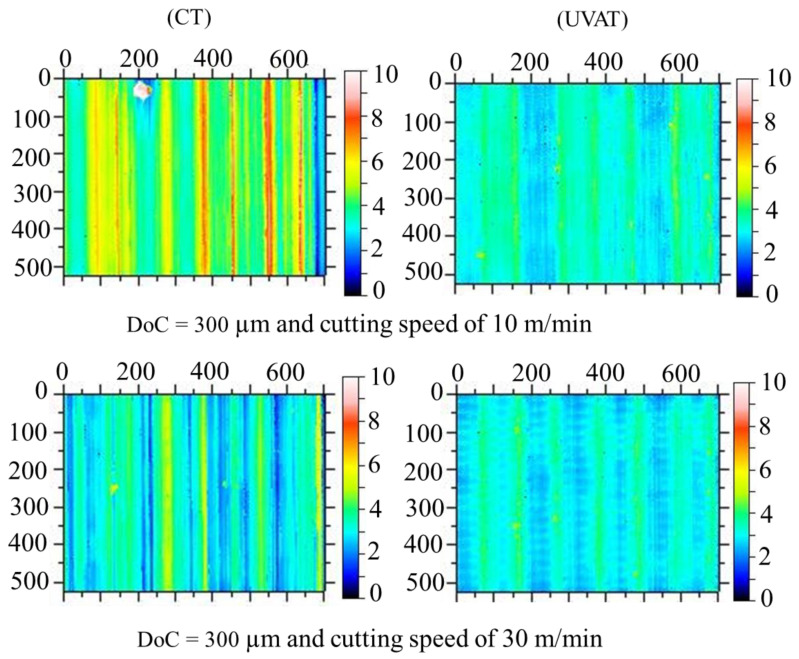
Optical scans of machined surfaces.

The quality of the product is assessed through many factors, but one of the most prominent factors is the surface topography of a machined part. A substantial enhancement in the machine surface quality was achieved in the UVAT process when judged with CT in the same cutting conditions as those presented in Figure 14. The *Ra* value measured at 30 m/min in CT was still higher when compared to that obtained during the UVAT process at 10 m/min. The tool movement in one complete vibration cycle produced an ironing effect on the turned surface, and a reduction of approximately 36–50% in the *Ra* was achieved at all of the tested conditions in the UVAT process when matched to CT.

**Figure 15:**
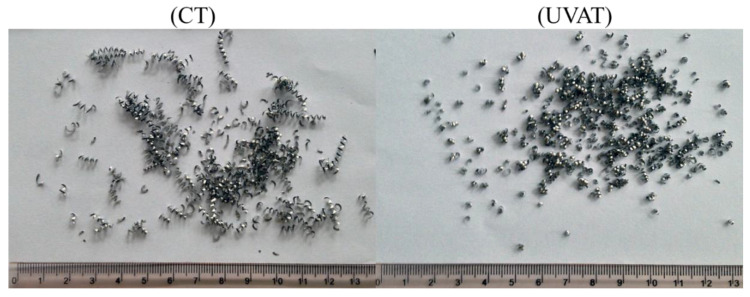
Chips of the studied alloy collected from the UVAT and CT processes.

The studied alloys resulted in discontinuous chips in the CT and UVAT processes in all of the tested conditions. The chips were analyzed for CRR and SA calculations. The chip size produced during UVAT was substantially smaller when matched to the chip produced during CT in the same cutting conditions. The discontinuity of the chips resulted from the addition of *Nd*, which has a lower melting temperature. The chip samples from the UVAT and CT processes are presented in Figure 15. 

The CCR was calculated using Equation (2), and SA was calculated using Equation (3) for the CT and UVAT processes [47].

(2) $$\mathrm{CCR}=\frac{H_{max}+H_{min}}{2d_{c}}.\;$$

(3) $$\mathrm{SA}={Tan}^{-1}(\frac{rcos\alpha }{1-rsin\alpha })$$

(4) $$r=\frac{t_{f}}{t_{o}}$$

where *H_max_* is the maximum height measured in serrated chips, the measured minimum height is denoted by *H_min_*, the net chip thickness is represented by *d_c_*, α is the rake angle of the cutting insert, which is 14.6° for the CP-500 inserts, *t_f_* is the thickness of the chips after machining, and *t_o_* is the initial chip thickness before machining.

**Figure 16:**
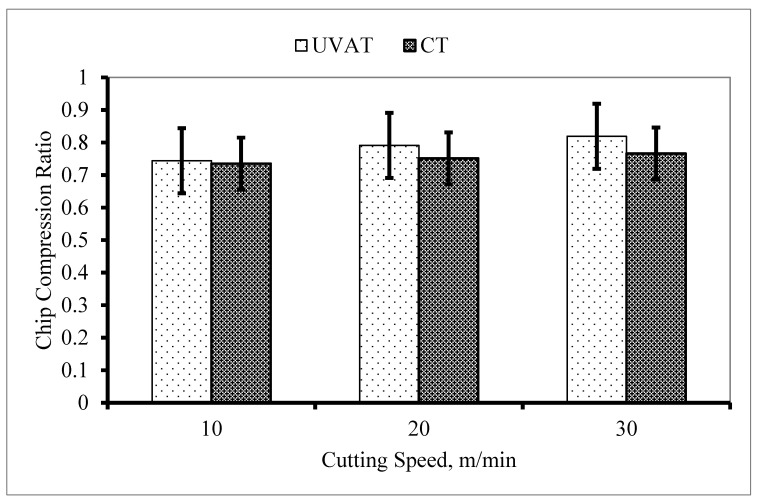
The calculated CCR at DoC = 300 µm.

The calculated CCR in the UVAT process was slightly higher than that of CT, as presented in Figure 16, and the possible reason for this is the vibration imposed on the cutting insert. A minor increase in the CCR was observed with an increase in cthe utting speed during both CT and UVAT, as expected

**Figure 17:**
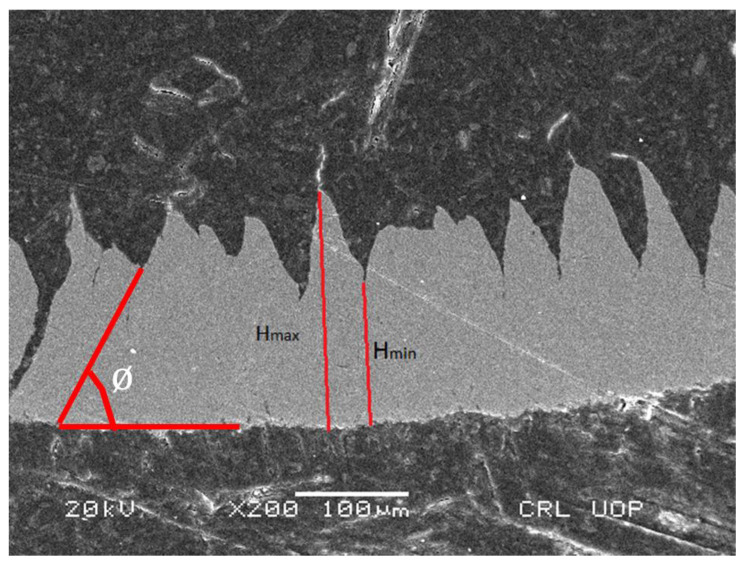
SEM image of the analyzed chip.

Similarly, the calculated SA for the CT and UVAT processes is shown in Figure 17. The results for the UVAT process showed a relatively higher magnitude of SA when compared to that of CT. An average SA of 86.2°, 81.2°, and 78.2° was measured at 10, 20, and 30 m/min, respectively, whereas in CT, the calculated SA was 84.8°, 78.9° and 73.6°, correspondingly. The increase of SA in the UVAT process can be attributed to the intermittent contact between the cutting insert and the chip. The vibro-impact phenomena resulted in more plastic deformation at the process zone when compared to CT. Additionally, the higher temperature level in the UVAT process also expedited the plastic deformation of the shear zone and resulted in a slight increase in SA. A minor decline in SA was noted with an increase in the cutting speed in the CT and UVAT processes.

### 4.3. Comparison of Simulated and Experimental Results

The data obtained from simulation of the developed algorithm are consistent with the experimental results for both of the studied processes. A comparative analysis of the simulation and experimental results is depicted in Table 6 and Table 7 for CT and UVAT, respectively. The predicted *Ft* and *Fr* in CT at 10 m/min velocity and DoC = 300 µm are 101.55 N and 60.25 N, respectively, whereas the tests led to an average value of approximately 101 N and 61 N, which are consistent with the simulation results. Similarly, the cutting forces predicted by the developed model for CT at all of the tested conditions are in good agreement with the experimental results (see Table 6).

Moreover, at DoC = 300 µm, the predicted maximum process zone temperature in CT was 257.74 °C at 10 m/min speed (see Table 6). The model predicted temperature levels of 333.00 °C and 366.66°C when the speed was increased to 20 m/min and 30 m/min, respectively. The experimental results showed temperatures of 262.00 °C, 325.00 °C, and 370.00 °C, correspondingly. The model predicted that the process zone temperature would have a maximum and minimum error of 3.11% and 0.71%, respectively, as shown in Figure 6.

Furthermore, the developed fuzzy model for CT predicted CCR values of 0.5838, 0.6464, and 0.7394 at 10 m/min speed studied DoC. The experimental results were measured, and the results showed that the model predicted the CCR with an accuracy of 99%. The simulations and experimental results of CCR are presented in Table 6.

The predicted and measured *Ra* value of the machined surface using CT is presented in Table 6. The fuzzy model for CT predicted the *Ra* value with a maximum error of 10%, which is still in the acceptable range. The developed model is a good alternative to predict the machine surface quality in CT. Furthermore, the SA predicted by the model is also in good agreement with the calculated results, and corresponding maximum and minimum errors of 8.5% and 0.05% were noted.

The developed fuzzy model for the UVAT process was used to predict the cutting forces, maximum process zone temperature, surface roughness, CCR, and SA, as presented in Table 7. At 10 m/min speed, the predicted *Ft* at 100, 200, and 300 µm DoCs were 12.8 N, 24.0 N, and 36.0 N, respectively. The experimental validation demonstrated an error of 6.25%, 4.16%, and 5.55%, correspondingly. Similarly, when the speed was increased, a gradual increase in the cutting forces was observed in UVAT both experimentally and through the fuzzy model. However, the overall error was less than 10% in the predicted forces and showed the acceptability of the developed model for cutting force estimations.

The predicted *Fr* by the simulations at all of the tested conditions awere5.76, 15.60, 26.20, 10.30, 20.89, 31.40, 18.24, 31.50, and 41.27 N, as presented in Table 7, whereas the measured *Fr* were 5, 14, 26, 10, 21, 35, 18, 32, and 42 N, correspondingly, and were shown to be in good agreement with the force level predicted by the fuzzy model.

The model was used for the prediction of the maximum process zone temperature during the UVAT process. The model predicted the temperature of the process zone at all of the tested cutting conditions, with a maximum difference of approximately 5 °C (Max 1.7% error) and are in good agreement with the experimental results.

The predicted *Ra* of 0.253 µm was obtained from the simulation model at 10 m/min speed and 100 µm DoC. A gradual increase of 0.132 µm and 0.171 µm in *Ra* was obtained at 200 µm and 300 µm DoCs, respectively, when compared to Ra achieved at 100 µm, and kept the speed intact, whereas the experiments resulted in *Ra* of 0.263 µm, 0.381 µm, and 0.424 µm, correspondingly. In addition, the fuzzy model is sensitive to cutting speed and showed surface roughness improvements with speed. The model also predicted significant improvements in the surface finish when compared to CT. The model results in a *Ra* of a machined surface with a minimum accuracy of approximately 96% in the UVAT process. 

The collected chips at 10 m/min speed and the studied DoCs (100 µm, 200 µm and 300 µm) resulted in a CCR of 0.64, 0.72, and 0.74, respectively, while, the fuzzy model predicted a CCR 0.712, 0.724, and 0.749, correspondingly. Additionally, at 20 m/min, the CCR predicted by the simulation were 0.75 and 0.79, respectively, at 200 µm and 300 µm DoC. The experiments yielded 0.68 and 0.79 at the same cutting conditions. Likewise, at 30 m/min and 300 µm DoC, a minimal error of 0.5% and 0.25% was recorded between experimental and simulation results, respectively. The SA results predicted by the simulation model are in good agreement with the experimental results, and the model can be used to predict SA at the tested cutting conditions with an accuracy of 98.5% as presented in Table 7.

## 5. Conclusions

This paper reported the development of fuzzy-based simulation algorithms to predict the cutting forces, process zone temperature, surface quality of a machined specimen, CCR, and SA in the CT and UVAT processes. The algorithms were simulated effectively, and the results were in good agreement with the achieved experimental results. The simulation model for UVAT showed a significant reduction in the cutting forces and improvements in the surface quality when compared to the CT process. The developed model for the CT and UVAT processes can be used effectively to calculate the cutting forces, temperature of the process zone, surface quality, CCR, and SA of the studied alloys at various testing conditions within the acceptable range of accuracy (approximately 94%). Additionally, these models can be helpful to avoid the extensive and costly experimental methods needed to estimate output machining parameters. These algorithms may require expert knowledge but can be good alternative for future industries and for the selection of cutting parameters in current industries.

## Figures and Tables

**Table 1:** The contribution of researchers using fuzzy logic tools in machining processes in recent years.

Process	Workpiece	Machining Input Parameter	Investigation	Reference
DP	Al-5083	V, FR	SQ, hole size	[20]
EDM	SS-304	pulse-on/off times and current	MRR and EWR	[22]
LM	PMMA	V, power of laser, CO_2_ pressure, and stand-off distance	Width-kerf	[23]
MP	Al-6061 T6	V, DoC, and FR	SQ	[24]
TP	AISI 1045	V, DoC, and FR and approach angle	SQ, TW and, MRR	[25]
MP	Al-6061 T6	Nozzle pressure, nozzle angle, and nanoparticle concentration	Cutting forces, cutting temperature, and SQ	[26]
TP	Al-Si-Cu-Fe and doped alloys	FR, V, and alloy type	SQ	[27]
AWJM	Brittle materials	Nozzle diameter, pressure of liquid, mass flow rate of abrasive jet traverse rate	DoC	[28]
TP	Ti64	V, DoC, FR,	SQ, TW, and specific cutting pressure	[29]
EDM drilling	AISI-1010 Steel	Voltage gap, spark ratio deviation, and change in the deviation in spark-ratio	Current, voltage, and ignition delay time	[30]
Wire EDM	Die steel	Thickness, FR, and corner angle	Machining accuracy at corner parts	[31]
EDM	Die steel	Discharge current, the polarity of the workpiece, voltage at open discharge, pulse-on time, duty factor,	MRR and electrode wear ratio	[32]
EDM	Tool steel	Powder concentration, open-circuit voltage, duty cycle, pulsed duration, pulsed peak current, powder concentration, powder size	Dimensional accuracy and precision	[33]
DP	AISI-1018 steel	V and FR	Process zone temperature, burr formation, and chip morphology	[34]
LAJEM	WC-Co composite	Inter-electrode gap, supply voltage, electrolyte concentration, and duty cycle	Hole taper angle, MRR, and SQ	[35]
TP	AISI-1015 steel	V, DoC, FR, coolant flow rate	TW and SQ	[36]
TP	ZrSiO4-grade-LM25 matrix composites	Coolant, V, FR, DoC	Cutting forces, SQ, and TW	[37]

Drilling process (DP), abrasive waterjet machining (AWJM), turning process (TP), electrical discharge machining (EDM), milling process (MP), laser machining (LM), speed (V), feed rate (FR), depth-of-cut (DoC), material removal rate (MRR), electrode wear rate (EWR), laser-assisted jet electro-chemical machining (LAJEM), surface quality (SQ), tool wear (TW).

**Table 2:** Input and output parameters used in the CT fuzzy inference system.

MF Type	Fuzzy Input Variables					Fuzzy Output Variables												
	Variable	Speed (v)		Depth-of-Cut (DoC)		Variable	Tangential Cutting Forces (*Ft*)		Radial Force Component (*Fr*)		Temperature (T)		Surface Roughness (Ra)		Chip Compression ratio (CCR)		Shear Angle (SA)	
		Parameter	Range	Parameter	Range		Parameter	Range	Parameter	Range	Parameters	Range	Parameter	Range	Parameter	Range	Parameter	Range
Triangular	VVL	[10 10 12.5]	[10 30]	[100 100 125]	[100 300]	EL	[33 33 38]	[33 103]	[23 23 25.71]	[23 61]	[195 195 207.5]	[195 370]	[0.297 0.297 0.3372]	[0.297 0.86]	[0.58 0.58 0.5933]	[0.58 0.766]	[48 48 50.57]	[48 84]
	VL	[10 12.5 15]		[100 125 150]		VVVL	[33 38 43]		[23 25.71 28.43]		[195 207.5 220]		[0.297 0.3372 0.3774]		[0.58 0.5988 0.6066]		[48 50.57 53.14]	
	L	[12.5 15 17.5]		[125 150 175]		VVL	[38 43 48]		[25.71 28.43 31.14]		[207.5 220 232.5]		[0.3372 0.3774 0.4176]		[0.5966 0.6066 0.6199]		[50.57 53.14 55.71]	
	ML	[15 17.5 20]		[150 175 200]		VL	[43 48 53]		[28.43 31.14 33.86]		[220 232.5 245]		[0.3774 0.4176 0.4578]		[0.6066 0.6199 0.6332]		[53.14 55.71 58.28]	
	M	[17.5 20 22.5]		[175 200 225]		L	[48 53 58]		[31.14 33.86 36.57]		[232.5 245 257.5]		[0.4176 0.4578 0.498]		[0.6199 0.6332 0.6465]		[55.71 58.28 60.85]	
	MH	[20 22.5 25]		[200 225 250]		MLL	[53 58 63]		[33.86 36.57 39.28]		[245 257.5 270]		[0.4578 0.498 0.5382]		[0.6332 0.6465 0.6598]		[58.28 60.85 63.42]	
	H	[22.5 25 27.5]		[225 250 275]		ML	[58 63 68]		[36.57 39.28 42]		[257.5 270 282.5]		[0.498 0.5382 0.5784]		[0.6465 0.6598 0.6731]		[60.85 63.42 66]	
	VH	[25 27.5 30]		[250 275 300]		M	[63 68 73]		[39.28 42 44.71]		[270 282.5 295]		[0.5382 0.5784 0.6186]		[0.6598 0.6731 0.6864]		[63.42 66 68.57]	
	VVH	[27.5 30 30]		[275 300 300]		MH	[68 73 78]		[42 44.71 47.43]		[282.5 295 307.5]		[0.5784 0.6186 0.6588]		[0.6731 0.6864 0.6997]		[66 68.57 71.13]	
						MHH	[73 78 83]		[44.71 47.43 50.14]		[295 307.5 320]		[0.6186 0.6588 0.699]		[0.6864 0.6997 0.713]		[68.57 71.13 73.7]	
						H	[78 83 88]		[47.43 50.14 52.85]		[307.5 320 332.5]		[0.6588 0.699 0.7392]		[0.6997 0.713 0.7263]		[71.13 73.7 76.27]	
						VH	[83 88 93]		[50.14 52.85 55.57]		[320 332.5 345]		[0.699 0.7392 0.7794]		[0.713 0.7263 0.7396]		[73.7 76.27 78.84]	
						VVH	[88 93 98]		[52.85 55.57 58.28]		[332.5 345 357.5]		[0.7392 0.7794 0.8196]		[0.7263 0.7396 0.7529]		[76.27 78.84 81.41]	
						VVVH	[93 98 103]		[55.57 58.28 61]		[345 357.5 370]		[0.7794 0.8196 0.86]		[0.7396 0.7529 0.766]		[78.84 81.41 84]	
						EH	[98 103 103]		[58.28 61 61]		[357.5 370 370]		[0.8196 0.86 0.86]		[0.7529 0.766 0.766]		[81.41 84 84]	

**Table 3:** Input and output parameters used in the UVAT fuzzy inference system.

MF Type	Fuzzy Input Variables					Fuzzy Output Variables												
	Variable	Speed (v)		Depth-of-Cut (DoC)		Variable	Tangential Cutting Forces (*Ft*)		Radial Force Component (*Fr*)		Temperature (T)		Surface Roughness (Ra)		Chip Compression ratio (CCR)		Shear Angle (SA)	
		Parameter	Range	Parameter	Range		Parameter	Range	Parameter	Range	Parameters	Range	Parameter	Range	Parameter	Range	Parameter	Range
Triangular	VVL	[10 10 12.5]	[10 30]	[100 100 125]	[100 300]	EL	[12 12 15]	[12 54]	[5 5 7.65]	[5 42]	[224 224 237.2]	[224 408]	[0.121 0.121 0.143]	[0.121 0.429]	[0.71 0.71 0.7173]	[0.71 0.8122]	[64 64 65.57]	[64 86]
	VL	[10 12.5 15]		[100 125 150]		VVVL	[12 15 18]		[5 7.65 10.3]		[224 237.2 250.4]		[0.121 0.143 0.165]		[0.70 0.7173 0.7246]		[64 65.57 67.14]	
	L	[12.5 15 17.5]		[125 150 175]		VVL	15 18 21]		[7.65 10.3 12.95]		[237.2 250.4 263.6]		[0.143 0. 165 0.187]		[0.7173 0.7246 0.7319]		[65.57 67.14 68.71]	
	ML	[15 17.5 20]		[150 175 200]		VL	[18 21 24]		[10.3 12.95 15.6]		[250.4 263.6 276.8]		[0.165 0.187 0.209]		[0.7246 0.7319 0.7392]		[67.14 68.71 70.28]	
	M	[17.5 20 22.5]		[175 200 225]		L	[21 24 27]		[12.95 15.6 18.25]		[263.6 276.8 290]		[0.187 0.209 0.231]		[0.7319 0.7392 0.7465]		[68.71 70.28 71.85]	
	MH	[20 22.5 25]		[200 225 250]		MLL	[24 27 30]		[15.6 18.25 20.9]		[276.8 290 303.2]		[0.209 0.231 0.253]		[0.7392 0.7465 0.7538]		[70.28 71.85 73.42]	
	H	[22.5 25 27.5]		[225 250 275]		ML	[27 30 33]		[18.25 20.9 23.55]		[290 303.2 316.4]		[0.231 0.253 0.275]		[0.7465 0.7538 0.7611]		[71.85 73.42 75]	
	VH	[25 27.5 30]		[250 275 300]		M	[30 33 36]		[20.9 23.55 26.2]		[303.2 316.4 329.6]		[0.253 0.275 0.297]		[0.7538 0.7611 0.7684]		[73.42 75 76.57]	
	VVH	[27.5 30 30]		[275 300 300]		MH	[33 36 39]		[23.55 26.2 28.85]		[316.4 329.6 342.8]		[0.275 0.297 0.319]		[0.7611 0.7684 0.7757]		[75 76.57 78.13]	
						MHH	[36 39 42]		[26.2 28.85 31.5]		[329.6 342.8 356]		[0.297 0.319 0.341]		[0.7684 0.7757 0.783]		[76.57 78.13 79.7]	
						H	[39 42 45]		[28.85 31.5 34.15]		[342.8 356 369.2]		[0.319 0.341 0.363]		[0.7757 0.783 0.7903]		[78.13 79.7 81.27]	
						VH	[42 45 48]		[31.5 34.15 36.8]		[356 369.2 382.4]		[0.341 0.363 0.385]		[0.783 0.7903 0.7976]		[79.7 81.27 82.84]	
						VVH	[45 48 51]		[34.15 36.8 39.45]		[369.2 382.4 395.6]		[0.363 0.385 0.407]		[0.7903 0.7976 0.8049]		[81.27 82.84 84.41]	
						VVVH	[48 51 54]		[36.8 39.45 42]		[382.4 395.6 408]		[0.385 0.407 0.429]		[0.7976 0.8049 0.8122]		[82.84 84.41 86]	
						EH	[51 54 54]		[39.45 42 42]		[395.6 408.8 408]		[0.407 0.429 0.429]		[0.8049 0.8122 0.8122]		[84.41 86 86]	

**Table 4:** The rules used in the UVAT simulation.

	Input		Output							Input		Output					
Rules No	Speed	Depth-of-Cut	Tangential Cutting Force	Radial Cutting Force	Temperature	Surface Roughness	Chip Compression Ratio (CCR)	Shear Angle (SA)	Rules No	Speed	Depth-of-Cut	Tangential Cutting Force	Radial Cutting Force	Temperature	Surface Roughness	Chip Compression Ratio (CCR)	Shear Angle (SA)
R1	VVL	VVL	EL	EL	EL	ML	EL	MH	R2	VVL	VL	VVVL	VVVL	VVVL	M	EL	MHH
R3	VVL	L	VVL	VVL	VVVL	MHH	VVVL	H	R4	VVL	ML	VL	VL	VVL	VH	VVVL	VVH
R5	VVL	M	L	L	VL	VVH	VVL	VVVH	R6	VVL	MH	MLL	MLL	L	VVVH	VVL	VVVH
R7	VVL	H	ML	ML	MLL	VVVH	L	VVVH	R8	VVL	VH	M	M	MLL	EH	MLL	EH
R9	VVL	VVH	MH	MH	ML	EH	MLL	EH	R10	VL	VVL	VVVL	VVVL	VVVL	MLL	VVVL	MHH
R11	VL	VL	VVL	VVL	VVVL	M	VVL	H	R12	VL	L	VL	VL	VVL	MHH	VVL	VH
R13	VL	ML	L	L	VL	VH	VL	VVH	R14	VL	M	MLL	MLL	L	VH	VL	VVVH
R15	VL	MH	ML	ML	MLL	VVH	VL	VVVH	R16	ML	H	M	M	MLL	VVH	L	EH
R17	VL	VH	MH	MH	ML	VVVH	MLL	EH	R18	VL	VVH	MHH	MHH	M	EH	MLL	EH
R19	L	VVL	VVL	VVL	VVL	MLL	VVL	H	R20	L	VL	VL	VL	VVL	ML	VL	H
R21	L	L	L	L	VL	M	VL	VH	R22	L	ML	MLL	MLL	L	H	L	VVVH
R23	L	M	ML	ML	MLL	H	L	VVVH	R24	L	MH	M	M	ML	VH	MLL	VVVH
R25	L	H	MH	M	M	VH	MLL	EH	R26	L	VH	MHH	M	MH	VVVH	ML	EH
R27	L	VVH	H	MHH	MH	EH	ML	EH	R28	ML	VVL	VVL	VVL	VVL	L	EL	L
R29	ML	VL	VL	VL	VL	M	VVVL	MLL	R30	ML	L	VL	VL	L	MH	VVL	ML
R31	ML	ML	L	L	MLL	MHH	VL	M	R32	ML	M	MLL	MLL	MLL	H	L	MH
R33	ML	MH	ML	ML	ML	H	ML	MH	R34	ML	H	M	M	M	H	MH	MHH
R35	ML	VH	M	MH	MH	VVH	MHH	H	R36	ML	VVH	MHH	MHH	MHH	VVVH	H	H
R37	M	VVL	VL	VVL	L	VL	EL	MLL	R38	M	VL	L	VL	L	L	VVL	ML
R39	M	L	MLL	L	MLL	ML	VL	M	R40	M	ML	ML	MLL	MLL	M	L	MH
R41	M	M	M	ML	ML	MHH	ML	MHH	R42	M	MH	MH	M	M	H	M	MHH
R43	M	H	MHH	MH	MH	H	MHH	H	R44	M	VH	H	MHH	MHH	VH	H	VH
R45	M	VVH	VH	H	H	VVH	VH	VH	R46	MH	VVL	L	VL	L	VL	VVVL	ML
R47	MH	VL	L	VL	MLL	L	VVL	M	R48	MH	L	MLL	L	ML	L	VL	MH
R49	MH	ML	ML	MLL	ML	ML	MLL	MHH	R50	MH	M	M	ML	M	M	ML	H
R51	MH	MH	MH	MH	MH	MH	MH	H	R52	MH	H	MHH	MHH	MHH	MHH	H	VH
R53	MH	VH	H	MHH	H	H	VH	VVH	R54	MH	VVH	VH	VH	VH	VVH	VH	VVH
R55	H	VVL	L	VL	L	VVL	VVVL	EL	R56	H	VL	MLL	VL	L	VVL	VVL	VVVL
R57	H	L	ML	L	MLL	VL	VL	VL	R58	H	ML	M	MLL	ML	L	L	MLL
R59	H	M	M	ML	M	MLL	L	ML	R60	H	MH	MH	M	MH	M	ML	ML
R61	H	H	MHH	MH	H	MH	M	M	R62	H	VH	H	MHH	VH	MH	H	MH
R63	H	VVH	VH	VH	VVH	VH	VH	MH	R64	VH	VVL	L	L	ML	VVVL	VVL	VVVL
R65	VH	VL	L	L	ML	VVVL	VL	VVL	R66	VH	L	MLL	MLL	M	VVVL	L	MLL
R67	VH	ML	M	ML	MH	VVL	L	ML	R68	VH	M	MH	MH	MH	VL	MLL	M
R69	VH	MH	MHH	MH	H	ML	M	MH	R70	VH	H	H	H	VH	M	MH	MH
R71	VH	VH	VH	VH	VVH	MHH	H	MHH	R72	VH	VVH	VVH	VVH	VVVH	H	VVVH	MHH
R73	VVH	VVL	MLL	MLL	ML	EL	VVL	EL	R74	VVH	VL	ML	ML	M	VVVL	VL	VL
R75	VVH	L	MH	M	MH	VVL	MLL	MLL	R76	VVH	ML	MHH	MHH	MH	VL	M	M
R77	VVH	M	H	H	MHH	L	MHH	MH	R78	VVH	MH	VH	VH	VH	MLL	H	MH
R79	VVH	H	VVH	VVH	VVH	M	VH	MHH	R80	VVH	VH	VVVH	VVVH	VVVH	MH	VVH	H
R81	VVH	VVH	EH	EH	EH	MHH	EH	H									

**Table 5:** The rules defined for the CT fuzzy inference system.

Input			Output						Input			Output					
Rules	Speed	Depth of Cut	Tangential Cutting Force	Radial Cutting Force	Temperature	Surface Roughness	Chip Compression Ratio	Shear Angle	Rules	Speed	Depth of Cut	Tangential Cutting Force	Radial Cutting Force	Temperature	Surface Roughness	Chip Compression Ratio	Shear Angle
R1	VVL	VVL	VVVL	EL	EL	VL	EL	L	R2	VVL	VL	VL	VVL	VVVL	MLL	VVL	ML
R3	VVL	L	MLL	L	VVL	ML	L	MH	R4	VVL	ML	M	ML	VVL	M	MLL	H
R5	VVL	M	MH	M	VL	MH	ML	VH	R6	VVL	MH	MHH	MHH	VL	H	MH	VH
R7	VVL	H	VH	VH	L	VH	H	VVH	R8	VVL	VH	VHHH	VVVH	MLL	VVVH	VH	VVVH
R9	VVL	VVH	EH	EH	MLL	EH	VVH	EH	R10	VL	VVL	EL	EL	VVVL	VVL	VVVL	MLL
R11	VL	VL	VL	VVL	VVL	L	VVL	MH	R12	VL	L	ML	L	VL	MLL	L	H
R13	VL	ML	ML	ML	VL	ML	ML	VH	R14	VL	M	MH	MH	L	M	M	VVH
R15	VL	MH	MHH	MHH	L	MHH	MHH	VVH	R16	ML	H	VH	VH	MLL	H	H	VVVH
R17	VL	VH	VVVH	VVVH	ML	VVH	VH	VVVH	R18	VL	VVH	EH	EH	ML	VVVH	VVH	EH
R19	L	VVL	VVVL	VVVL	VVL	VVL	L	L	R20	L	VL	VVL	VL	VL	VL	MLL	L
R21	L	L	VL	MLL	VL	L	ML	MLL	R22	L	ML	ML	M	L	ML	ML	ML
R23	L	M	M	MH	MLL	M	M	M	R24	L	MH	MHH	MHH	M	MH	MHH	MH
R25	L	H	H	VH	MH	H	H	MHH	R26	L	VH	VH	VVH	MHH	VH	VH	H
R27	L	VVH	VVH	VVVH	H	VVH	VVH	VH	R28	ML	VVL	VVVL	VVVL	VVL	VVL	ML	L
R29	ML	VL	VVL	VL	VL	VL	ML	L	R30	ML	L	VL	L	L	L	ML	MLL
R31	ML	ML	ML	ML	MLL	ML	M	ML	R32	ML	M	M	M	ML	M	M	M
R33	ML	MH	MHH	MHH	M	MHH	MH	MH	R34	ML	H	H	H	MH	H	MHH	MHH
R35	ML	VH	VH	VHH	MHH	VVH	VH	H	R36	ML	VVH	VVH	VVVH	H	VVVH	VVH	VH
R37	M	VVL	VVVL	VVVL	VL	VL	M	VVL	R38	M	VL	VL	VL	L	L	M	L
R39	M	L	L	MLL	MLL	MLL	MH	ML	R40	M	ML	ML	M	ML	ML	MH	M
R41	M	M	M	MH	M	M	MH	MHH	R42	M	MH	MHH	MH	MH	MH	MHH	MHH
R43	M	H	VH	H	MHH	MHH	VH	H	R44	M	VH	VVH	VVH	H	VH	VVH	VH
R45	M	VVH	VVH	VVVH	VH	VVH	VVVH	VVH	R46	MH	VVL	EL	EL	L	L	MH	VVVL
R47	MH	VL	VVL	VVL	MLL	ML	MH	VL	R48	MH	L	L	L	ML	M	MHH	MLL
R49	MH	ML	ML	ML	M	MH	MHH	M	R50	MH	M	M	M	MH	MHH	MHH	MH
R51	MH	MH	MH	MH	MH	H	H	MHH	R52	MH	H	VH	H	H	VH	VH	H
R53	MH	VH	VVH	VVH	VH	VVH	VVH	H	R54	MH	VVH	VVVH	VVVH	VH	VVVH	VVVH	VH
R55	H	VVL	EL	EL	MLL	VVL	M	VVL	R56	H	VL	VVVL	VL	ML	VL	M	L
R57	H	L	L	L	M	L	MH	ML	R58	H	ML	ML	ML	MHH	MLL	MH	M
R59	H	M	M	M	MHH	ML	MH	MH	R60	H	MH	MHH	MHH	H	ML	MHH	MHH
R61	H	H	H	H	VH	M	H	H	R62	H	VH	VVH	VVH	VVH	MH	VH	VH
R63	H	VVH	VVVH	VVVH	VVH	MH	VVH	VVH	R64	VH	VVL	VVVL	EL	MLL	VVVL	MH	VVVL
R65	VH	VL	VL	VVL	M	VVL	MH	VL	R66	VH	L	L	L	MH	VL	MH	L
R67	VH	ML	ML	ML	MHH	L	MHH	ML	R68	VH	M	M	ML	H	L	MHH	M
R69	VH	MH	MHH	MH	VH	MLL	H	MH	R70	VH	H	H	H	VVH	ML	VH	MHH
R71	VH	VH	VH	VVH	VVH	M	VVH	H	R72	VH	VVH	VVVH	EH	VVVH	M	VVVH	VH
R73	VVH	VVL	EL	EL	ML	EL	MHH	EL	R74	VVH	VL	VVL	VVL	M	VVVL	MHH	VVL
R75	VVH	L	L	L	MHH	VVL	H	L	R76	VVH	ML	MLL	ML	H	VL	H	MLL
R77	VVH	M	ML	ML	VH	VL	H	ML	R78	VVH	MH	MH	MH	VH	L	VH	M
R79	VVH	H	H	H	VVH	MLL	VVH	MH	R80	VVH	VH	VH	VVH	VVVH	M	VVVH	MHH
R81	VVH	VVH	VVH	EH	EH	MH	EH	H									

**Table 6:** Comparative analysis of simulation and experimental results in CT.

Cutting Conditions		Simulation Results						Experimental Results					
Speed (m/min)	DOC (µm)	*Ft* (N)	*Fr* (N)	T_max_ (°C)	*Ra* (µm)	CCR	SA (°)	*Ft* (N)	*Fr* (N)	T_max_ (°C)	*Ra* (µm)	CCR	SA (°)
10	100	38.00	23.78	198.00	0.419	0.5838	58.2	38 ± 5	23 ± 4	195	0.413 ± 0.036	0.58 ± 0.15	58.59 ± 10
10	200	68.00	39.32	220.14	0.583	0.6464	73.7	72 ± 7	43 ± 3	227	0.621 ± 0.038	0.65 ± 0.20	77.56 ± 08
10	300	101.55	60.25	257.74	0.851	0.7394	83.3	101 ± 6	61 ± 5	262	0.861 ± 0.050	0.735 ± 0.08	84.88 ± 07
20	100	38.00	25.72	232.66	0.419	0.6730	53.1	35 ± 4	24 ± 3	231	0.401 ± 0.050	-	-
20	200	63.00	42.00	270.34	0.542	0.6860	66.0	68 ± 5	45 ± 2	276	0.601 ± 0.040	0.68 ± 0.20	71.67 ± 09
20	300	93.00	58.38	333.00	0.787	0.7530	78.8	94 ± 6	58 ± 3	325	0.783 ± 0.050	0.75 ± 0.10	78.89 ± 06
30	100	34.44	23.78	270.34	0.308	0.6996	48.7	34 ± 3	23 ± 3	269	0.297 ± 0.060	-	-
30	200	63.00	39.32	333.00	0.419	0.7128	63.4	64 ± 3	40 ± 4	326	0.419 ± 0.047	0.71 ± 0.17	63.46 ± 12
30	300	93.00	60.25	366.60	0.624	0.7622	73.7	93 ± 5	59 ± 5	370	0.611 ± 0.046	0.77 ± 0.10	73.67 ± 05

**Table 7:** Comparative analysis of experimental and simulation results in UVAT.

Cutting Conditions		Simulation Results						Experimental Results					
Speed (m/min)	DOC (µm)	*Ft* (N)	*Fr* (N)	T_max_ (°C)	*Ra* (µm)	CCR	SA (°)	*Ft* (N)	*Fr* (N)	T_max_ (°C)	*Ra* (µm)	CCR	SA (°)
10	100	12.8	5.76	227.0	0.253	0.712	76.57	12 ± 3.7	05 ± 3.0	224	0.263 ± 0.02	0.65 ± 0.16	76.98 ± 8
10	200	24.0	15.60	263.5	0.385	0.724	84.42	23 ± 2.6	14 ± 4.0	261	0.381 ± 0.01	0.72 ± 0.15	85.46 ± 3
10	300	36.0	26.20	303.2	0.424	0.749	85.54	38 ± 2.4	26 ± 3.2	302	0.424 ± 0.02	0.74 ± 0.10	86.20 ± 4
20	100	21.0	10.30	263.6	0.188	0.712	71.85	20 ± 1.7	10 ± 3.0	261	0.195 ± 0.02	-	-
20	200	33.0	20.89	303.0	0.319	0.753	78.10	33 ± 2.2	21 ± 2.0	303	0.313 ± 0.01	0.68 ± 0.15	78.30 ± 6
20	300	45.0	31.40	355.9	0.385	0.796	81.28	46 ± 3.2	35 ± 4.0	355	0.392 ± 0.02	0.79 ± 0.14	81.20 ± 6
30	100	27.0	18.24	303.2	0.127	0.725	64.50	28 ± 4.2	18 ± 3.2	298	0.121 ± 0.02	-	-
30	200	42.0	31.50	342.8	0.209	0.774	76.60	41 ± 2.3	32 ± 2.0	340	0.219 ± 0.02	0.77 ± 0.12	76.70 ± 4
30	300	53.1	41.27	404.4	0.319	0.818	79.70	54 ± 3.1	42 ± 3.8	408	0.327 ± 0.02	0.82 ± 0.18	78.50 ± 6
